# Spatially Resolved Optoelectronic Properties of Al-Doped Zinc Oxide Thin Films Deposited by Radio-Frequency Magnetron Plasma Sputtering Without Substrate Heating

**DOI:** 10.3390/nano10010014

**Published:** 2019-12-19

**Authors:** Eugen Stamate

**Affiliations:** National Centre for Nano Fabrication and Characterization, Technical University of Denmark, Ørsteds Plads, 2800 Kongens Lyngby, Denmark; eust@dtu.dk

**Keywords:** transparent conducting oxides, aluminum doped zinc oxide, magnetron plasma sputtering

## Abstract

Transparent and conducting thin films were deposited on soda lime glass by RF magnetron sputtering without intentional substrate heating using an aluminum doped zinc oxide target of 2 inch in diameter. The sheet resistance, film thickness, resistivity, averaged transmittance and energy band gaps were measured with 2 mm spatial resolution for different target-to-substrate distances, discharge pressures and powers. Hall mobility, carrier concentration, SEM and XRD were performed with a 3 mm spatial resolution. The results reveal a very narrow range of parameters that can lead to reasonable resistivity values while the transmittance is much less sensitive and less correlated with the already well-documented negative effects caused by a higher concentration of oxygen negative ions and atomic oxygen at the erosion tracks. A possible route to improve the thin film properties requires the need to reduce the oxygen negative ion energy and investigate the growth mechanism in correlation with spatial distribution of thin film properties and plasma parameters.

## 1. Introduction

Transparent and conductive thin films are important for a large number of applications, including but not limited to: touch screens, solar cells, smart windows (low-e, chromogenic devices) and light emitting diodes [1,2,3,4,5]. Oxides doped with metals, generically known as transparent conductive oxides (TCO) are successfully used nowadays, with indium tin oxide (ITO) being the best material, with a resistivity of around 10^−4^ Ωcm and transmittance above 88% [1]. However, the high demand for large area applications, coupled with the reduced abundance of indium, limits market penetration for very large area applications, such as low-e windows and solar cells [3,4]. This motivation sustains intensive research on alternative materials, with aluminum-doped zinc oxide (AZO) being one of the most promising choice due to the high abundance of Zn and Al [2]. For example, cost effective solar cells based on Cu (In,Ga)Se_2_ (CIGS) and Cu_2_ZnSnS_4_ (CZTS) absorbers have been fabricated with a TCO based on AZO [6,7]. There are several methods used to deposit AZO, including physical vapor deposition (under various operation conditions for magnetron sputtering, such as radio-frequency [8,9,10,11,12,13,14,15,16,17,18,19,20], medium-frequency [8,21,22,23,24,25,26], DC [8,16,22,26,27,28,29,30,31,32,33,34,35,36,37], pulsed DC [38], high power impulse [39,40], ion beam assisted [41], chemical vapor deposition [1,5] and other chemical methods such as spin coating and sol gel [2,4]. Among them, magnetron plasma sputtering has been successfully used to deposit ITO on large area substrates (up to 15 m^2^) and is also regarded as a viable and cost effective solution for AZO [1,2,3,4,5]. However, the resistivity of AZO thin films is about 5 to 10 times higher than that of ITO, with better values only for limited locations on the substrate [2,5]. The main reason for this is the electronegativity of oxygen that easily forms negative ions by attaching low-energy electrons emitted from the target by secondary emission or generated by plasma [2,5]. Since the sputtering target builds up a negative bias (positive ions produce the sputtering after being accelerated in a thin space charge layer named the plasma sheath), the negative ions [42] are accelerated over the sheath towards the substrate, and assist the film growth with energies distributed from 0 to 500 eV for operation in DC and 0–300 eV for operation in radio-frequency discharge, as recently reported by Ellmer et al. [43,44]. The presence of permanent magnets behind the target, so as to produce a high-density plasma close to the surface, results in a non-uniform erosion. This non-uniformity is correlated with the radial distribution of the negative ions that is eventually mirrored on the substrate [45]. At the same time, one expects a non-uniform distribution of the oxygen released from the target (mainly for short target-to-substrate distances) which can also influence the thin film growth [5]. Up to date, both energetic negative ions and oxygen distribution are considered as the main reasons responsible for the poor optoelectronic properties of AZO over the substrate surface [2,5,45]. AZO deposited by magnetron plasma sputtering was, and yet is, intensively studied, with a large number of works reporting resistivity values in the range of 10^−4^ Ωcm only for small substrate areas or for films with transmittance below 80% [1,2,3,4,5]. The lowest resistivity for AZO, of 8.54 × 10^−5^ Ωcm, was reported by pulsed laser deposition over a non-specified area [46]. However, a critical investigation reveals that further improvement of AZO properties could only be possible by simultaneously achieving a resistivity below 3 × 10^−4^ Ωcm and a transmittance above 88%, for a substrate area comparable with the target area. If these optoelectronic properties are achieved without intentional substrate heating, then such a process can be used to coat heat sensitive substrates.

The aim of this work is to identify the best range of deposition parameters (pressure, power and target-to-substrate distance) and to provide a set of carefully measured spatial distribution profiles for optoelectronic properties (sheet resistance, resistivity, mobility, carrier concentration and transmittance) of AZO thin films that can be used as a reference for further studies so as to eventually understand the thin film growth mechanism.

## 2. Materials and Methods

**Figure 1:**
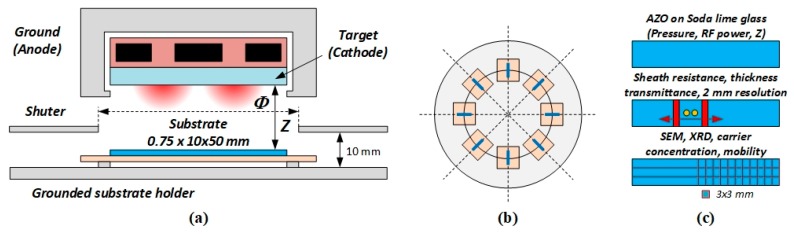
(**a**) Schematic of the experimental setup, (**b**) samples arrangement on the substrate holder, (**c**) sample characterization.

Clean soda lime glass samples of 10 × 50 mm and 0.75 mm in thickness were used as substrates to deposit AZO thin films by a two-inch in diameter Zinc/Alumina (ZnO/Al_2_O_3_, 97/2 wt%) target (Kurt Lesker, Jefferson Hills, PA, USA) mounted on a TORUS^®^ cathode powered in radio-frequency at 13.56 MHz (see Figure 1a).

The vacuum chamber was large enough (50 cm in diameter) to accommodate 8 samples at the same time, placed on a large holder that could rotate so as to expose the samples one by one, to different discharge conditions (power, *P*_RF_, pressure, *p*, and target-to-substrate distance, *Z*), without turning off the discharge (see Figure 1b). A large disk shutter was used to prevent deposition on samples during the time needed to adjust the discharge parameters. The deposition was done by aligning a *Φ* = 60 mm in diameter opening in the shutter disk between the target and the substrate at a constant distance of 10 mm between the opening and the substrate holder. The target-to- substrate distance was adjusted by translating the cathode upwards. The discharge pressure was varied in the range of 1.4 to 50 mTorr, the RF power from 10 up to 100 W and target-to-substrate distance from 25 up to 100 mm. There was no intentional heating of the substrate except for a temperature rise due to plasma exposure, which was less than 70 degrees (measured with a no-contact FTX-100-LUX + OSENSA Innovations, Burnaby, BC, Canada, temperature transmitter) after 1 h deposition time for all process parameters presented in this work. The sheath resistance was measured with a resolution of 2 mm using a four-probe system, configured to accommodate the substrate dimensions (see Figure 1c). The thin film thickness was measured with a thin film analyzer Filmetrics F20 (San Francisco, CA, USA) and confirmed by SEM with the same spatial resolution as the sheet resistance so as to enable the calculation of the AZO thin film resistivity profile. The transmittance spectra was measured using an Agilent Cary 100 UV-Vis photo-spectrometer (Santa Clara, Ca, USA), in steps of 2 mm, to provide the averaged transmittance in the range of 400 to 700 nm and the band gap energy from Tauc’s plot. In the last steps of characterization, the samples were cut into 3 × 3 mm pieces as presented in Figure 1c and separate rows were used for SEM (Zeiss Merlin, Oberkochen, Germany), XRD and Hall effect measurement (ezHEMS from Nanomagnetics Instruments, Oxford, UK) of carrier concentration and mobility.

## 3. Results

**Figure 2:**
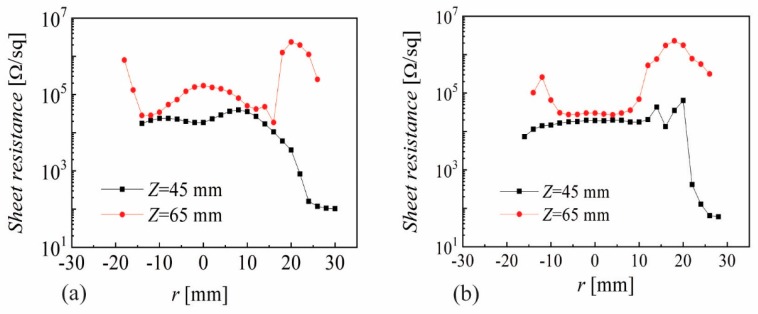
Sheet resistance as a function of radial position for (**a**) *p* = 1.4 mTorr and (**b**) *p* = 3 mTorr where *P*_RF_ = 20 W and deposition time 60 min.

The sheet resistance as a function of radial position (*r* = 0 at sample center) for *Z* = 45, and 65 mm, *P*_RF_ = 20 W and 60 min deposition time is presented in Figure 2a for *p* = 1.4 mTorr and (b) *p* = 3 mTorr, respectively, where 1.4 mTorr was the lowest pressure to sustain the plasma.

**Figure 3:**
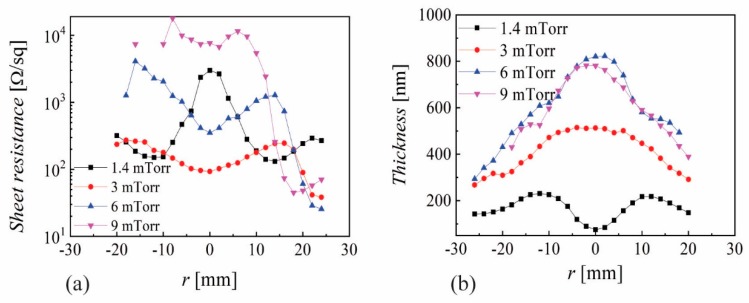
Spatial distribution of (**a**) sheet resistance and (**b**) film thickness for different pressures and *Z* = 35 mm and *P*_RF_ = 20 W.

Despite the small change in pressure, one can see a flattening of the sheet resistance for −10 < *r* < 10 mm when increasing the *p* from 1.4 to 3 mTorr. However, the most remarkable thing is the difference, of almost two orders of magnitude, for 20 < *r* < 30 mm at both pressures, as well as the variation of the sheet resistance with more than one order of magnitude for *Z* = 45 mm at the locations corresponding to *r* = 10 mm and *r* = 25 mm. It is important to note that the sheet resistance profiles are symmetric with respect to *r* = 0 mm and on purpose we used a translation of 4 mm so as to be able to capture thin film properties for 25 < *r* < 30 mm (close to the edge of the shutter). Figure 2a exhibits a good correlation with the erosion tracks for *Z* = 45 mm (two humps structure, for *r* ~ −10 mm and 10 mm respectively) while increasing *Z* to 65 mm gives a convolution, with a single hump for −10 < *r* < 10 mm. Such behavior was reported more than 20 years ago and was associated with the possible influence of negative ions or oxygen distribution [45]. Application wise, sheet resistance values above 100 Ω/sq were too high, and increasing Z led to even higher values, so that the target-to-substrate distance was further decreased. Figure 3a presents the sheath resistance and (b) the film thickness for different pressures and *Z* = 35 mm. In this case, the correlation with the erosion tracks is evident for *p* ≥ 3 mTorr, with two humps that are getting closer by increasing *p*. The spatial distribution at *p* = 1.4 mTorr shows a strong central peak, revealing that the plasma discharge exhibits a torch-like profile, resulting from an inefficient plasma production by the magnetic field at low pressures. However, it is remarkable to see that a very small change in pressure (from 1.4 to 3 mTorr) has a significant effect on the plasma (revealed by the film thickness) and sheet resistance profile. Once again, one can notice very large variations in the sheet resistance, as the decrease from 10^4^ Ω/sq to 350 Ω/sq, only by moving from *r* = 5 mm to *r* = 17 mm at *p* = 9 mTorr. The film thickness presented in Figure 3b helps one to understand that the torch-like discharge mode causes intensive re-sputtering on the sample surface for −10 < *r* < 10 mm, while higher pressures reveal almost parabolic profiles with some small shoulders correlated with the erosion track for 6 and 9 mTorr. A higher film thickness at 3 mTorr with respect to 1.4 mTorr suggests a significant change in plasma density within this narrow pressure change.

**Figure 4:**
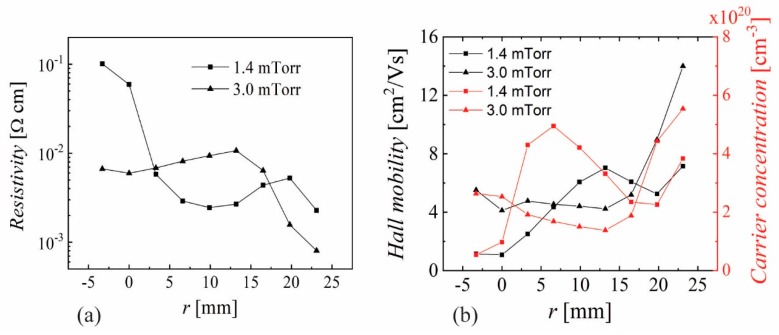
Spatial distribution of (**a**) resistivity and (**b**) mobility and carrier concentration for samples deposited at 1.4 and 3 mTorr, *Z* = 35 mm and *P*_RF_ = 20 W.

**Figure 5:**
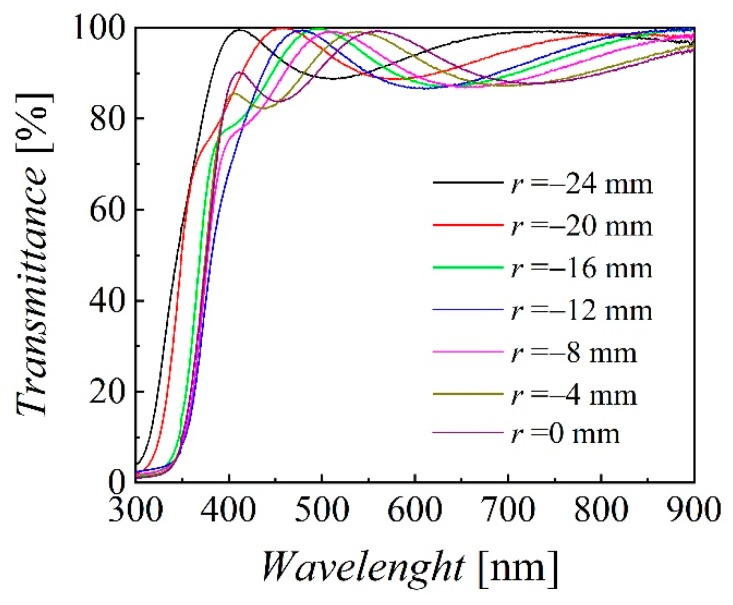
Transmittance spectra at different radial locations for *p* = 3 mTorr, *Z* = 35 mm and *P*_RF_ = 20 W.

**Figure 6:**
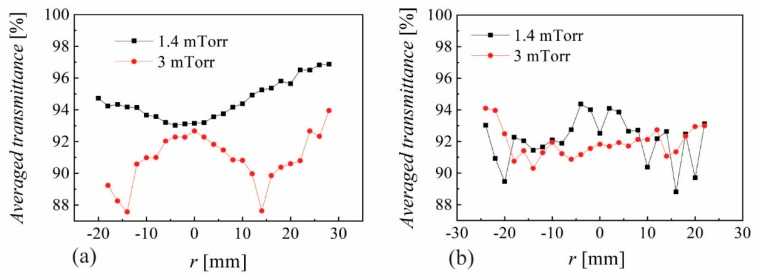
Spatial distribution of averaged transmittance for (**a**) *Z* = 35 mm and (**b**) *Z* = 45 mm where *P*_RF_ = 20 W.

The resistivity, Hall mobility and carrier concentration measured on 3 × 3 mm^2^ samples (see Figure 1c) are presented in Figure 4 for −5 < *r* < 25 mm and *p* = 1.4 and 3 mTorr, with lowest resistivity and highest mobility and carrier concentration at the edge (*r* = 23 mm) of the sample deposited at 3 mTorr. The optical performance is characterized by the transmittance spectra in the visible range that have been measured between 250 and 900 nm. As example, the radial distribution of transmittance spectra from *r* = −24 mm to the sample center are presented in Figure 5 for *p* = 3 mTorr, *Z* = 35 mm and *P*_RF_ = 20 W, from which one can see the interference oscillations correlated with the film thickness. The averaged transmittance (400 to 700 nm) for 1.4 mTorr and 3 mTorr is presented in Figure 6a for *Z* = 35 mm and (b) for *Z* = 45 mm, where an obvious correlation with the erosion track can be seen only for the sample at 3 mTorr and *Z* = 45 mm. All of the values are above 87%, even reaching above 93% over the whole sample deposited at 1 mTorr and *Z* = 45 mm, thus revealing that the main challenge is to reduce the resistivity.

**Figure 7:**
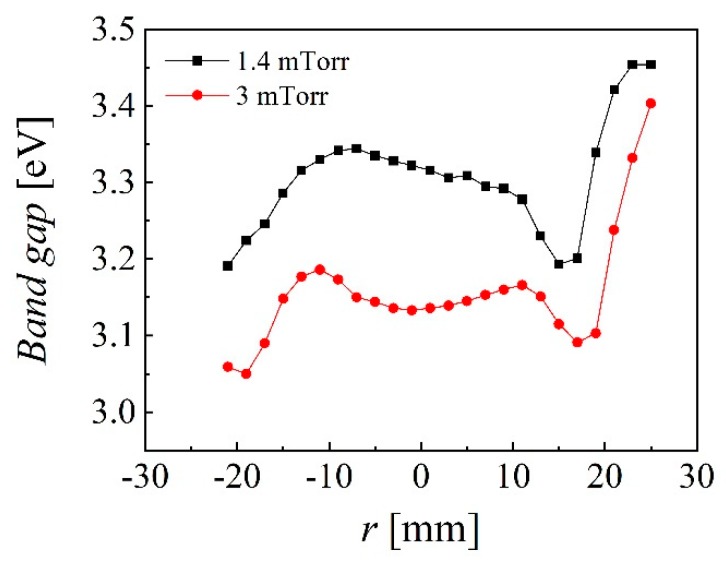
Spatial distribution of energy band gap for samples deposited at 1.4 and 3 mTorr where *Z* = 35 mm and *P*_RF_ = 20 W.

Another important parameter is the band gap with a theoretical value of 3.37 eV for ZnO and expected increase with up to 0.5 eV by Al doping. The band gap of samples deposited at 1.4 and 3 mTorr and *Z* = 35 mm (20 W, 60 min) were calculated using Tauc’s plot of transmittance spectra and are presented in Figure 7 with an evident correlation with the erosion tracks and also showing the highest values (above 3.4 eV) at the same locations with lowest resistivity values and highest mobility and carrier concentration.

**Figure 8:**
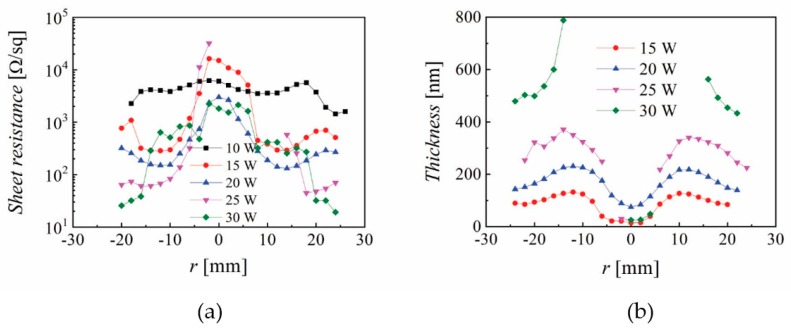
Spatial distribution for the (**a**) sheet resistance and (**b**) film thickness for different discharge powers (*P*_RF_) where *p* = 1.4 mTorr, *Z* = 35 mm and a 60 min deposition time.

The spatial distribution of the (a) sheet resistance and (b) film thickens is presented in Figure 8 for different discharge powers at 1.4 mTorr, *Z* = 35 mm and 60 min deposition time. While the central part (−10 < *r* < 10 mm) was significantly affected by the torch-like discharge, noticed also in Figure 3, the lowest sheet resistance values (below 50 Ω/sq) were measured at the edge. The deep film thickness near *r* = 0 mm is caused by re-sputtering on the substrate, with no measurable values for powers above 20 W. The sheet resistance, Hall mobility, carrier concentration, resistivity and averaged transmittance for data points in Figure 8 at *r* = 24 mm are presented in Table 1 and show the lowest resistivity of 5.45 × 10^−4^ Ωcm, 17.3 cm^2^/Vs for mobility, 6.63 × 10^20^ cm^−3^ for carrier concentration and 88% for averaged transmittance. Such values are indeed very good for several applications that need TCO’s with moderate properties, including low emissivity windows.

**Figure 9:**
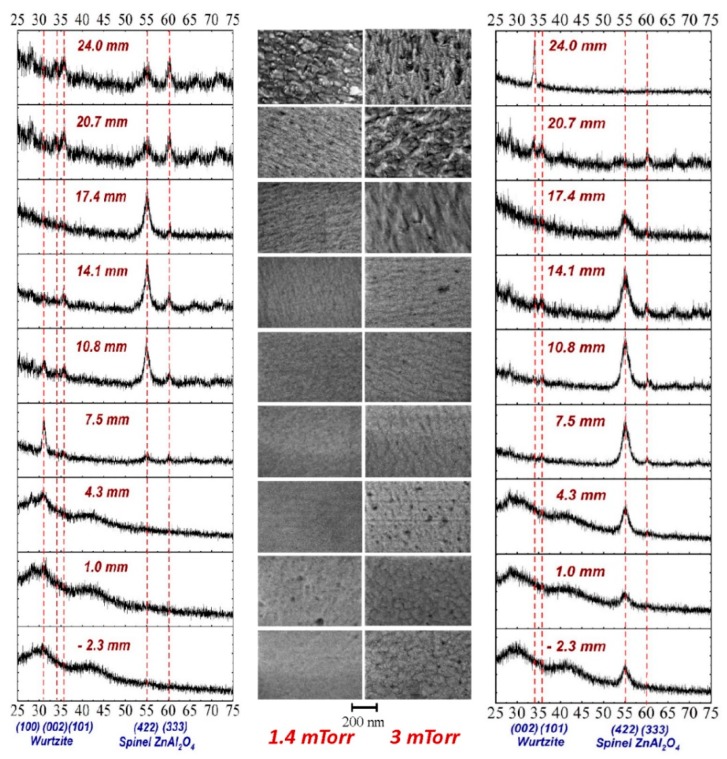
XRD and SEM performed on 3 × 3 m^2^ substrate pieces cut from samples deposited at 1.4 (left) and 3 mTorr (right) presented in Figure 3a (20 W, *Z* = 35 mm, 60 min).

However, the challenge remains to attain such values all over the substrate. XRD and SEM performed on the 3 × 3 m^2^ substrate pieces cut from the samples deposited at 1.4 (left) and 3 mTorr (right) presented in Figure 3a (20 W, *Z* = 35 mm, 60 min) are presented in Figure 9. Several crystalline structures can be identified with stronger peaks of Spinel (422) correlated with the erosion tracks and a well visible peak for Wurtzite (002) present only at the edge, corresponding to the lowest resistivity, as presented in Figure 4. Surface morphology by SEM reveals a higher roughness near the edge. Cross-SEM images have been presented elsewhere [20], including some showing a possible correlation with a transition in the Thornton diagram from zone 1 to zone 2 in a narrow pressure range.

## 4. Discussion

AZO by magnetron sputtering has been under investigation for more than 30 years, with several detailed reports examining its non-uniformity aspects [45]. While the focus for an extended period was on reporting record values for the lowest resistivity, it became evident in recent years that resistivity values below 10^−3^ Ωcm are very difficult to obtain over an area comparable with that of the sputtering target for averaged transmittance values above 88%. Significant effort was also devoted to understanding the growth mechanism in correlation with the possible role of oxygen negative ions and atomic oxygen distribution [43,44]. The results presented in Section 3 reveal a very narrow range for deposition parameters where one can obtain reasonably good resistivity (10^−3^ Ωcm, with lower values outside the zone mirrored by the erosion tracks on the substrate): low pressure (2–4 mTorr), short target-to-substrate distance (30–45 mm) and low RF power (20–35 W for a 2 inch target). Increasing the pressure above 4 mTorr produced higher resistivity values. A similar trend was observed by increasing the target-to-substrate distance (see Figure 2). The short distance (*Z*) and low pressure also limits the discharge power to below 30 W (higher powers result in significant re-sputtering at the central part of the sample). The correlation of resistivity with the erosion track is obvious (see Figure 3 and Figure 8) and it has been reported before. The XRD and SEM investigation (Figure 9) shows a Wurtzite (002) structure and larger grains only at the edge of the sample |*r*| > 20 mm while the Spinel (422) was dominant at locations facing the erosion tracks. Due to the glass substrate’s contribution, the EDX investigation gave no relevant trends [20]. In a more detailed recent investigation, it was concluded that Al content was directly correlated with compressive stress, and the spatial inhomogeneity and pressure dependence could be related to particle bombardment [20]. An important aspect presented in this work is the very large variations one can get (almost two orders in magnitude for resistivity) within a very small shift in location (5–10 mm). This suggests that any attempts to understand the growth mechanism while rotating the substrate or neglecting the role of the investigation location with respect to the erosion track cannot lead to meaningful conclusions. The possibility of obtaining better resistivity values in certain locations far from the erosion track is also known and was intentionally used in several configurations [2]. However, this approach will be difficult to be implemented in large area coatings. As shown in Figure 1a, the present results were obtained by depositing a 50 mm long sample through a 60 mm opening in the shutter. Combined with the rather short target-to-substrate distance, one can see a possible shadowing effect at the sample’s ends. The measurements performed without the shutter exhibited higher resistivity values all over the sample, a fact that suggests an additional positive role by placing a grounded electrode near the cathode [47]. The main point of this work is the optoelectronic characterization of the deposited films with a spatial resolution of 2–3 mm, which reveals the importance of trying to understand the growth mechanism by a careful examination of the entire spatial distribution of both the thin film and plasma parameters. While surface characterization techniques such as XPS, TOF-SIMS and XRD allows one to perform spatially resolved analytical investigations, this possibility alone cannot unveil the growth mechanism due to the need for coupling with spatially resolved plasma diagnostics. While electrostatic probes are subject to contamination and electromagnetic field distortions [48,49], optical diagnostics need complex 2D laser-induced fluorescence setups [50] to reveal the needed information.

## 5. Conclusions

Spatially resolved optoelectronic parameters of AZO thin films deposited by RF magnetron sputtering without intentional substrate heating were performed with the aim of narrowing down the process parameters that give the best film properties. For proper use in applications, both a low resistivity (below 3 × 10^−3^ Ωcm) and high transmittance (above 88%) should be attained on substrates comparable with target size. The strong correlation of film properties with the erosion tracks observed at a low pressure and a short target-to-substrate distance suggests that oxygen negative ions could be of higher relevance than the atomic oxygen concentration. A proper conclusion needs an adequate sputtering process design where one is able to control the negative ion energy.

## Figures and Tables

**Table 1:** Sheet resistance, Hall mobility, carrier concentration, resistivity and averaged transmittance for the data points in Figure 8 at *r* = 24 mm for different discharge powers.

RF Power [W]	Sheet Resistance/sq	Hall Mobility [cm^2^/Vs]	Carrier Concentration [cm^−3^]	Resistivity [cm]	Averaged Transmittance [%]
10	2320	2.62	5.07 × 10^19^	4.69 × 10^−2^	92.2
15	311	6.93	3.05 × 10^20^	2.95 × 10^−3^	89.6
20	175	7.15	3.84 × 10^20^	2.27 × 10^−3^	86.2
25	67.2	9.33	4.33 × 10^20^	1.54 × 10^−3^	87.7
30	13.6	17.3	6.63 × 10^20^	5.45 × 10^−4^	88.5
